# Relation of Heart Rate and its Variability during Sleep with Age, Physical Activity, and Body Composition in Young Children

**DOI:** 10.3389/fphys.2017.00109

**Published:** 2017-02-24

**Authors:** David Herzig, Prisca Eser, Thomas Radtke, Alina Wenger, Thomas Rusterholz, Matthias Wilhelm, Peter Achermann, Amar Arhab, Oskar G. Jenni, Tanja H. Kakebeeke, Claudia S. Leeger-Aschmann, Nadine Messerli-Bürgy, Andrea H. Meyer, Simone Munsch, Jardena J. Puder, Einat A. Schmutz, Kerstin Stülb, Annina E. Zysset, Susi Kriemler

**Affiliations:** ^1^Preventive Cardiology and Sports Medicine, University Clinic for Cardiology, Inselspital, Bern University Hospital, University of BernBern, Switzerland; ^2^Epidemiology, Biostatistics and Prevention Institute, University of ZurichZurich, Switzerland; ^3^Institute of Pharmacology and Toxicology, University of ZurichZurich, Switzerland; ^4^University Hospital of Child and Adolescent Psychiatry and Psychotherapy, University of BernBern, Switzerland; ^5^Zurich Center for Integrative Human Physiology, University of ZurichZurich, Switzerland; ^6^Endocrinology, Diabetes, and Metabolism Service, Centre Hospitalier Universitaire Vaudois (CHUV)Lausanne, Switzerland; ^7^Child Development Center, University Children's Hospital ZurichZurich, Switzerland; ^8^Children's Research Center, University Children's Hospital ZurichZurich, Switzerland; ^9^Department of Clinical Psychology and Psychotherapy, University of FribourgFribourg, Switzerland; ^10^Department of Psychology, University of BaselBasel, Switzerland; ^11^Division of Pediatric Endocrinology, Diabetology and Obesity, Centre Hospitalier Universitaire Vaudois (CHUV)Lausanne, Switzerland

**Keywords:** heart rate variability, children, cardiovascular health, cardiac autonomic nervous system, growth, SPLASHY

## Abstract

**Background:** Recent studies have claimed a positive effect of physical activity and body composition on vagal tone. In pediatric populations, there is a pronounced decrease in heart rate with age. While this decrease is often interpreted as an age-related increase in vagal tone, there is some evidence that it may be related to a decrease in intrinsic heart rate. This factor has not been taken into account in most previous studies. The aim of the present study was to assess the association between physical activity and/or body composition and heart rate variability (HRV) independently of the decline in heart rate in young children.

**Methods:** Anthropometric measurements were taken in 309 children aged 2–6 years. Ambulatory electrocardiograms were collected over 14–18 h comprising a full night and accelerometry over 7 days. HRV was determined of three different night segments: (1) over 5 min during deep sleep identified automatically based on HRV characteristics; (2) during a 20 min segment starting 15 min after sleep onset; (3) over a 4-h segment between midnight and 4 a.m. Linear models were computed for HRV parameters with anthropometric and physical activity variables adjusted for heart rate and other confounding variables (e.g., age for physical activity models).

**Results:** We found a decline in heart rate with increasing physical activity and decreasing skinfold thickness. HRV parameters decreased with increasing age, height, and weight in HR-adjusted regression models. These relationships were only found in segments of deep sleep detected automatically based on HRV or manually 15 min after sleep onset, but not in the 4-h segment with random sleep phases.

**Conclusions:** Contrary to most previous studies, we found no increase of standard HRV parameters with age, however, when adjusted for heart rate, there was a significant decrease of HRV parameters with increasing age. Without knowing intrinsic heart rate correct interpretation of HRV in growing children is impossible.

## Introduction

Cardiovascular (CV) risk factors develop in early childhood. They may alter autonomic balance which seems to be associated with reduced heart rate variability (HRV; Zhou et al., 2012; Vrijkotte et al., 2015). On the other hand, physical activity (PA) and a high exercise capacity appear to positively influence indices of HRV in children and adolescents by increasing HRV (Nagai et al., 2004; Brunetto et al., 2005; Gutin et al., 2005; Buchheit et al., 2007; Krishnan et al., 2009; Michels et al., 2013; Radtke et al., 2013). Parameters of HRV as a surrogate measure of autonomic nervous function have been found to be predictors for cardiovascular mortality in cardiovascular disease (Bigger et al., 1992; Fei et al., 1996; Dekker et al., 2000). Therefore, and due to the ease of application and its non-invasive nature, HRV has become popular in children for the assessment of cardiovascular health.

Previously performed studies in children have used short measurements of 5–10 min during the day (Michels et al., 2013; Seppälä et al., 2014; Gasior et al., 2015) or 24-h recordings (Silvetti et al., 2001; Zhou et al., 2012). Short-term recordings are often poorly standardized because children cannot relax and stay motionless on demand, while 24-h monitoring is highly dependent on the duration of physical activity, sleep, and resting (Montgomery-Downs et al., 2006). An appropriate recording condition could be provided by deep sleep (Brandenberger et al., 2005), a highly standardized state with low sympathetic activity, and stationary HRV data undisturbed by environmental stimulants. Despite these advantages, nighttime recordings have rarely been used in children (Finley and Nugent, 1995; Goto et al., 1997; Radtke et al., 2013), possibly due to the confounding by sleep depth (Villa et al., 2000). Although determination of deep sleep usually requires recording of an electroencephalogram (EEG) and traditional sleep staging, this was not possible in the present setting. Therefore, we developed our own algorithm to detect deep sleep phases.

Pediatric studies that have reported HRV data have generally reported an increase in HRV markers of vagal tone with increasing age up to age 6 (Finley and Nugent, 1995; Goto et al., 1997) or 10 years (Villa et al., 2000; Silvetti et al., 2001; Michels et al., 2013). However, all of these studies have also found an age associated decrease in heart rate (HR). It is questionable whether this decrease can simply be ascribed to an age associated increase in vagal tone or whether it may be related to the substantial growth of the heart during this age period. Some authors have suggested that failure to adjust for changing HR may over- or underestimate HRV responses (Sacha and Pluta, 2008; Billman, 2013; Monfredi et al., 2014). One recent study has adjusted HRV parameters measured in children aged 6–13 years with prevailing HR and has in fact found a decrease in HRV parameters with age (Gasior et al., 2015).

The main aim of the present study was to assess the effect of PA, anthropometric parameters and body composition on linear and non-linear HRV parameters while correcting for the growth associated decline of HR. A secondary aim was to compare different methodologies to measure HRV during sleep in young children.

## Materials and methods

### Study design and participants

The present study was conducted under the umbrella of the Swiss Preschoolers Health Study (SPLASHY, Current Controlled Trials Registry: ISRCTN41045021). SPLASHY is a prospective, multi-center, national study to investigate the effect of stress and physical activity on health in preschool children. The present study is based on cross-sectional data of the baseline assessments in 2014 including healthy preschoolers aged 2–6 years recruited from randomly selected childcare centers in Switzerland. Ethical approval was obtained from the responsible ethical committees of the respective cantons, and the children's parents provided written informed consent in accordance with the Declaration of Helsinki.

### Study procedure

The children were fitted with two chest electrodes and a small device (e-Motion, Mega Electronics, Kuopio, Finland) which records interbeat duration (R-R intervals) at a sampling rate of 1,000 Hz, and a three-dimensional accelerometer validated for preschool children (Pate et al., 2006; Actigraph wGT3x, Shalimar, FL, USA) around the waist. Children and parents/caregivers were instructed in how to continuously wear the monitor over 8 consecutive days except during swimming and showering. The parents were instructed in taking off and reinstalling the R-R interval monitor if children took a shower/bath and in taking it off in the morning following the night recording. Height and weight were measured based on standard procedures and body mass index (BMI) *z*-scores calculated according to World Health Organization (WHO) criteria (http://www.who.int/childgrowth/standards/bmi_for_age/en/). Skinfolds were measured in triplicate on the right body side at four sites (biceps, triceps, subscapular, suprailiac) using a Harpenden caliper. The average of the values for each site was calculated and the sum of the four sites was used for statistical analysis.

### HRV measurement

**Figure 1:**
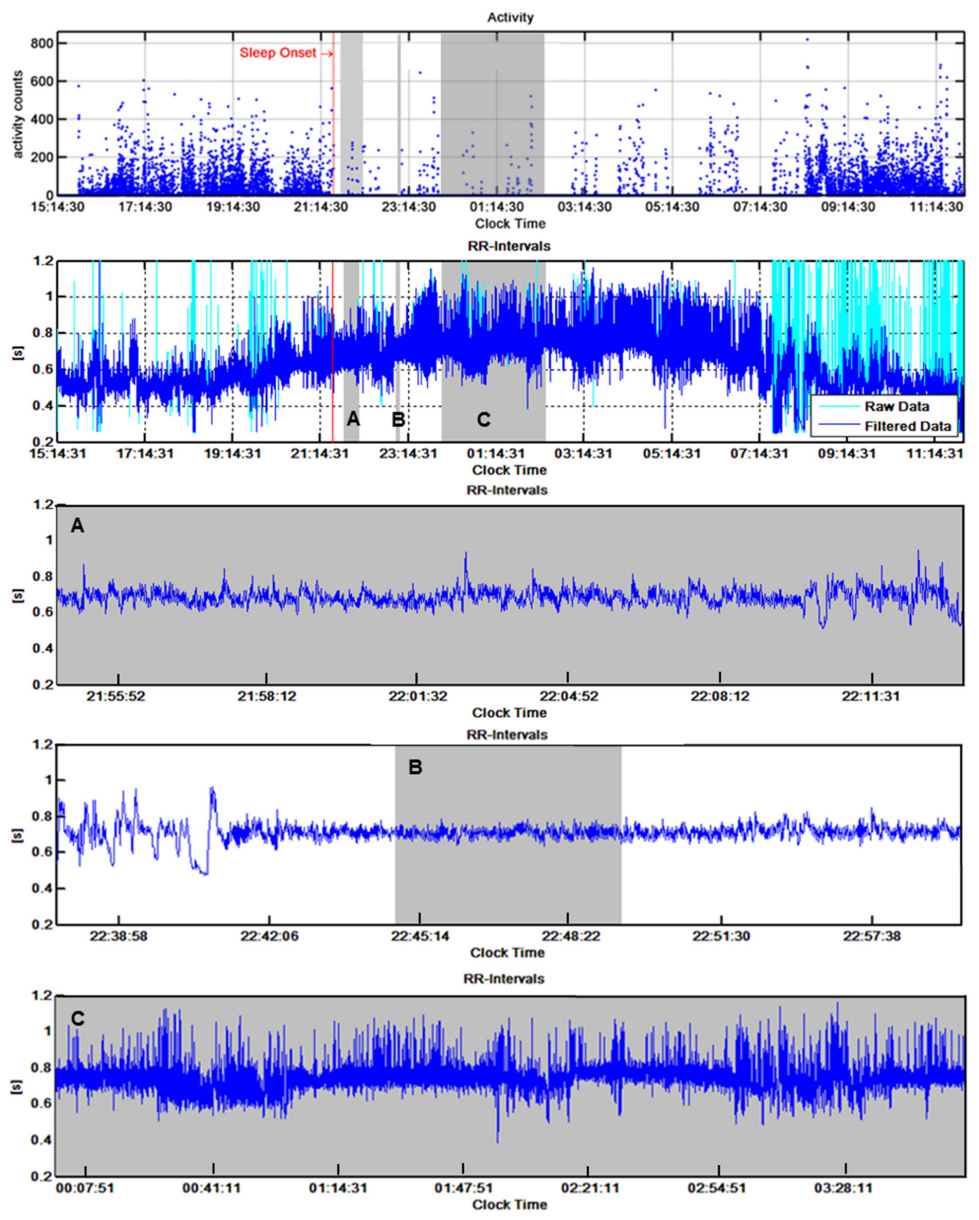
**Typical example of accelerometer (top panel) and R-R interval recordings (second panel from top) of one child**. The three bottom panels show the R-R intervals of the three selected segments as indicated by shading: the 15 min after sleep onset segment (*15'aSO*, A), the high % high frequency segment (high %HF, B), and the 4-h segment **(C)**. Please note that the time scales on the x-axes vary.

R-R intervals were further analyzed using Matlab (2014a, The Mathworks, Natick, MA) with a procedure developed specifically for this study. R-R interval recordings and accelerometer data of the same time period were synchronized (Figure 1). Deep sleep was approximated by means of two different approaches: firstly, by assuming that young children enter deep sleep within 20 min after sleep onset (Montgomery-Downs et al., 2006), and secondly, based on HRV parameters, since some studies have shown that determination of sleep stages by HRV showed close correspondence to EEG signals (Charloux et al., 1998; Otzenberger et al., 1998; Brandenberger et al., 2001). To allow comparison of our results with the methodology of existing studies who have collected HRV night data irrespective of sleep phase, we have also analyzed a 4-h segment between midnight and 4 a.m. Consequently, of the valid R-R interval recordings, three segments were determined for HRV analysis: (1) a 5-min segment during automatically determined deep sleep with highest percentage of high frequency (HF) power of the HRV frequency spectrum (*high %HF*); (2) a 20-min segment during the first deep sleep phase starting 15 min after sleep onset *(15'aSO)*; and (3) a 4-h segment starting at midnight (*4-h*).

#### 15'aSO

This 20-min time segment was selected 15 min after sleep onset (SO). SO times were automatically determined for each night based on (1) no accelerometer activity and (2) a clear shift toward a lower HR. When no simultaneous valid accelerometer recording was available, SO was determined based on the sudden constant decrease in HR only. Two researchers from the team visually and independently validated the R-R interval signal from the automatically computed SO times. If the automatic SO detection was obviously wrong, the SO time was changed manually. In a further step, all manually adjusted SO times and unclear data were discussed in a group of four researchers and a decision was made by agreement of all. ECG signal artifacts (i.e., due to removal of the device or loss of electrodes during sleep) and data files with unidentifiable SO time were excluded.

#### High %HF

By means of a custom built Matlab procedure, percentage HF power of total power (TP) (for explanation see chapter “HRV analysis” below) was calculated from 5-min windows moved by 30 s over the whole night. Previous studies have shown specific HRV characteristics in deep sleep with stationary and uncorrelated successive R-R intervals in deep sleep and a high percentage of HF power (Brandenberger et al., 2005; Shinar et al., 2006), which has been confirmed also in children (Villa et al., 2000; Ferri et al., 2002). We developed an algorithm to identify the first segment with HF exceeding 90% of TP during a minimum of 10 min, which was chosen for HRV analysis. If no segment was found using a threshold of 90%, the threshold was lowered to 80% if necessary (<10% of the recordings). A 5-min segment was placed in the middle of the identified segment and used for the HRV analysis.

#### 4-h segment

A *4-h* segment was selected from 12 to 4 a.m. regardless of SO time or sleep/wake phases, in analogy to previous studies (Finley and Nugent, 1995; Goto et al., 1997) for comparative reasons.

### HRV analysis

The following time domain parameters were used for analysis: HR (beats.min^−1^), the square root of the mean squared differences of adjacent R-R intervals (RMSSD, ms) and the standard deviation of all R-R intervals (SDNN, ms). For spectral analysis, R-R intervals were interpolated using a cubic spline interpolation method and then resampled at 4 Hz. We applied an advanced smoothness prior approach for detrending of R-R intervals with a smoothing parameter of λ = 500, which corresponds to a cut-off frequency of 0.035 Hz (Tarvainen et al., 2002). We used an artifact correction algorithm that eliminated R-R intervals in case of deviations of 30% or more of adjacent R-R intervals and replaced them using a cubic-spline interpolation. Power spectral density was then calculated using Fast Fourier Transformation. Frequency domain parameters were total power (TP, ms^2^, 0–0.4 Hz), low-frequency power (LF, ms^2^, 0.04–0.15 Hz), high-frequency power (HF, ms^2^, 0.15–0.4 Hz), and the LF/HF power ratio (Camm, 1996). HF and LF in normalized units are not reported due to their redundancy with LF/HF power ratio. Markers for vagal tone are generally accepted to be RMSSD and HF (Camm, 1996). Additionally, detrended fluctuation analysis (DFA) was performed (Rajendra Acharya et al., 2006). The short-term fractal scaling exponent alpha 1 was calculated for window sizes between 4 and 11 beats and used for analysis.

### Physical activity

Accelerometery data was recorded at a sampling rate of 30 Hz. Periods of 20 min of continuous zero values were interpreted as not worn and removed. A minimum of 4 days with 10 h of wearing time on each day were required for inclusion in the data analysis. PA data recorded between 7 a.m. and 9 p.m. were included in the analysis that defined total daily PA (counts.min^−1^), light PA (LPA, min.day^−1^), moderate-to-vigorous PA (MVPA, min.day^−1^), vigorous PA (VPA, min.day^−1^), total PA (TPA, min.day^−1^), and sedentary time (ST, min.day^−1^) using the cutpoints by Butte et al. (2014).

### Statistical analysis

Statistical analysis was performed using the software R (Version 3.2.3, R Core Team, 2015). Normality of the data was visually assessed using QQ-plots. Differences between boys and girls in anthropometric parameters and physical activity were assessed using unpaired *t-*tests. HRV parameters of the three segments were compared pair-wise using Wilcoxon signed-rank testing. We decided not to use Friedman's test for repeated measures because of the list-wise omission of cases when one segment was missing. Spearman correlations were performed for corresponding HRV parameters of the different segments. Effects of body composition and physical activity on HRV parameters of all three segments were assessed using linear regression models. Analyses were performed for dependent variables HR, lnRMSSD, lnSDNN, and DFA alpha 1 and independent anthropometric parameters entered together with HR, as well as activity parameters entered together with HR and age. Non-normally distributed HRV variables were log transformed. Models were tested with regard to satisfaction of underlying statistical assumptions such as normal distribution of residuals and homoscedasticity. A Spearman correlation matrix with all variables entered into the models was performed to assess the potential presence of collinearity and suppressor effects. A *p* < 0.05 was considered statistically significant. A sub-analysis was performed on the basis of nine sub-groups: Children were divided into three approximately equally sized (n ≅ 103) age groups (young: 2–3.49 years; mid: 3.5–4.19 years; old: 4.2–6 years) as well as three equally sized HR groups (low: <83 beats.min^−1^; mid 83–91 beats.min^−1^; high: >91 beats.min^−1^). Of the nine resulting groups, the smallest was selected (*n* = 27). From the remaining groups, 27 children were randomly selected within each group. Kruskal–Wallis tests were performed between age groups for all HRV parameters. Also, Kruskal–Wallis-tests were performed within each age group between HR groups and within each HR group between age groups.

## Results

### Study population

We collected data from 476 children attending 84 different childcare centers in Switzerland. Of these, 402 children had overnight ECG measurements of which 325 could be analyzed. Valid accelerometry data were obtained from 435 children. Only children with both, valid overnight ECG and valid daytime accelerometry data, i.e., 309 children, were included in the statistical analysis. The sample included 162 boys and 147 girls with a mean age of 3.9 ± 0.7 years, height of 102.8 ± 6.6 cm, weight of 17.1 ± 2.5 kg, BMI *z*-score of 0.5 ± 0.9, and sum of skinfolds of 25.8 ± 5.5 mm. Total PA was 1428 ± 284 cpm, children spent 236 ± 53, 457 ± 47, 76 ± 35, 5 ± 5 min.day^−1^ in sedentary time, light, moderate-to-vigorous, and vigorous PA, respectively. Boys were taller and heavier, had a smaller sum of skinfolds and a greater volume of TPA and MVPA than girls (all *p* ≤ 0.02), while age, BMI, LPA, VPA, and sedentary time did not differ between sexes. Children's anthropometric characteristics as well as physical activity data for the different age groups are shown in Table 1.

**Table 1:** **Anthropometric, physical activity and HRV data for the different age groups**.

	**2 years**	**3 years**	**4 years**	**5 years**
N [m,f]	19 (5; 14)	158 (58; 49)	108 (58; 50)	23 (13; 10)
Age [years]	2.8 (2.6; 2.9)	3.5 (3.3; 3.7)	4.3 (4.1; 4.6)	5.5 (5.2; 5.9)
Height [cm]	94 (91; 95)	100 (97; 107)	106 (103; 108)	117 (110; 118)
Weight [kg]	14.1 (12.9; 15.8)	16.0 (15.0; 17.5)	17.8 (16.2; 18.9)	20.9 (18.8; 22.9)
BMI [kg.m^−2^]	16.4 (15.7; 17.1)	16.1 (15.4; 16.9)	15.9 (15.3; 16.6)	15.6 (15.0; 16.8)
TPA [counts.min^−1^]	1,321 (1,102; 1,401)	1,357 (1,160; 1,554)	1,489 (1,289; 1,680)	1,571 (1,367; 1,705)
MVPA [min.day^−1^]	54.8 (35.6; 78.2)	60.6 (45.0; 87.4)	83.6 (62.0; 110.0)	101.7(84.3; 114.3)
HR [beats.min^−1^]	87.6 (85.2; 93.26)	86.9 (80.7; 93.4)	84.9 (78.0; 90.2)	80.0 (74.1; 85.8)
RMSSD [ms]	62.3 (36.5; 86.5)	56.7 (35.6; 94.8)	57.3 (37.8; 85.2)	66.0 (38.9; 124.8)
SDNN [ms]	58.9 (32.6; 72.1)	47.2 (31.6; 75.6)	49.9 (33.3; 69.3)	53.5 (32.0; 95.2)
HF [ms^2^]	2,526 (754; 3,699)	1,499 (630; 3,983)	1,559 (719; 3,292)	2,051 (718; 6,309)
LF [ms^2^]	228 (172; 464)	242 (102; 579)	230 (111; 620)	415 (144; 723)
LF/HF	0.14 (0.08; 0.27)	0.16 (0.10; 0.25)	0.16 (0.10; 0.30)	0.17 (0.10; 0.26)
Total Power [ms^2^]	2,961 (902; 4,538)	1,764 (742; 4,652)	1,284 (869; 3,961)	2,474 (824; 7,684)
DFA alpha 1	0.60 (0.52; 0.69)	0.55 (0.46; 0.63)	0.55 (0.44; 0.63)	0.52 (0.41; 0.59)

*TPA, total physical activity; MVPA, moderate-to-vigorous physical activity; HR, heart rate; RMSSD, square root of the mean squared differences of adjacent RR intervals; SDNN, standard deviation of RR intervals; HF, high frequency power; LF, low frequency power; DFA, detrended fluctuation analysis*.

*Data are presented as median (interquartile range). HRV parameters are calculated from the high %HF segments*.

### Comparison of the three segments

Out of 309 measured children, 309 *15'aSO* segments could be analyzed. One *high %HF* segment could not be detected due to loss of functioning of the ECG-monitor early in the night and 308 nights could be used for this method. Thirty-three *4-h* segments had to be excluded due to the absence of valid ECG data. HRV parameters analyzed of the three segments are summarized in Table 2. Spearman correlation coefficients of at least 0.9 were found for HR, RMSSD and SDNN between the *15'aSO* and the *high %HF* segments (all *p* < 0.001). Despite these high correlations, a significant difference was found for each HRV parameter (*p* < 0.01) due to a small systematic difference (Table 2), congruent with a lower HR in the *high %HF* segment (−1.8 ± 4.3%) compared to the *15'aSO* segment. Also, the *high %HF* segment appeared on average 35 ± 51 min later during the course of the night. Spearman correlation coefficients between the *4-h* segments and both other segments were all smaller than 0.6 (all *p* < 0.001).

**Table 2:** **Median (IQR) of HRV parameters of the different nighttime segments**.

	**Segment 1 15-min-aSO**	**Segment 2 High-HF%**	**Segment 3 4-h**	**Difference between segment 1 and 2 [%]**
HR [beats.min^−1^]	87.4 (81.7; 93.4)	85.7 (79.5; 91.7)^a^	85.6 (80.3; 85.6)	−1.9
RMSSD [ms]	57.4 (37.2; 87.9)	58.5 (39.9; 91.2)^a^	61.5 (42.8; 85.0)	1.9
SDNN [ms]	53.2 (37.2; 72.7)	49.4 (32.5; 72.5)^a^	86.2 (68.0; 106.3)^a^^,^^b^	−7.1
HF power [ms^2^]	1,558 (658; 3,324)	1,721 (705; 3,691)^a^	1,936 (985; 3,729)^a^	10.5
LF power [ms^2^]	405 (231; 947)	250 (106; 608)^a^	1,320 (816; 2,036)^a^^,^^b^	−38.1
LF/HF	0.25 (0.15; 0.45)	0.16 (0.10; 0.27)^a^	0.68 (0.49; 0.93)^b^	−36.0
Total Power [ms^2^]	2,259 (993; 4,397)	2,028 (826; 4,599)^a^	7,366 (5,759; 9,625)^a^^,^^b^	−10.2
DFA alpha 1	0.58 (0.46; 0.69)	0.55 (0.45; 0.64)	0.62 (0.47; 0.81)	−5.2%

IQR, interquartile range; HRV, heart rate variability; HR, heart rate; aSO, after sleep onset; RMSSD, square root of the mean squared differences of adjacent RR intervals; SDNN, standard deviation of the RR intervals; HF, High frequency power; LF, low frequency power; DFA, detrended fluctuation analysis;

a p < 0.01 (Wilcoxon signed-rank test) for difference with segment 1;

b *p > 0.01 (Wilcoxon signed-rank test) for difference with segment 2*.

### HRV parameters and effects of age, PA, and body composition

**Figure 2:**
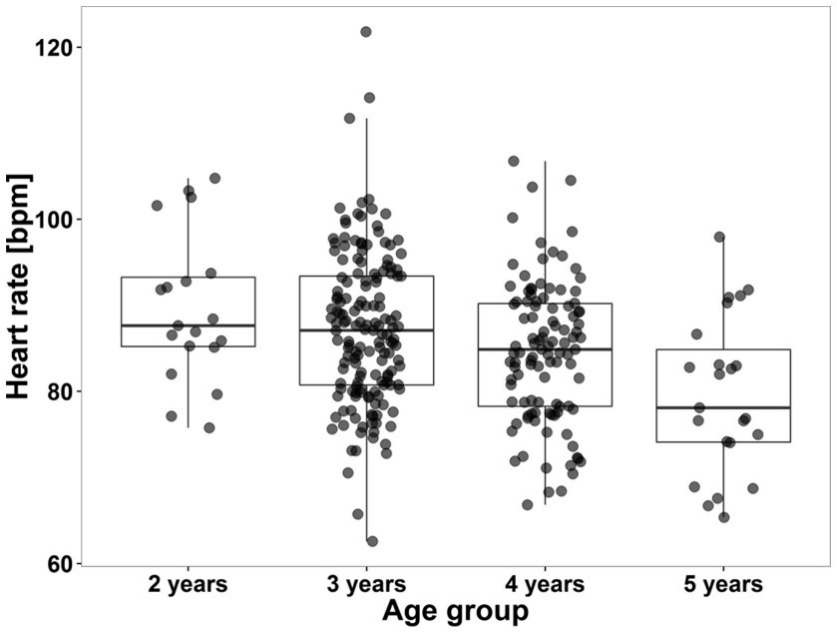
**Boxplot showing “deep sleep” heart rates of children according to their age**. Box plots show group medians (solid line), IQR (box outline) and spread of data without outliers (whiskers) for each group. The spread of dots with regard to the x-axis reflects younger and older children within each age group. There was a significant difference according to Kruskal–Wallis testing between children aged 2 and 5 years (*H* = 17.8, *p* < 0.001). Parametric linear regression revealed a significant decline of heart rate with increasing age (*r* = −0.27, *p* < 0.001). Data are based on HRV data from the *high %HF* segments.

HRV data from the *high %HF* segments for the different age groups are reported in Table 1. HR of the *high %HF* segments for each age group is shown in Figure 2.

**Figure 3:**
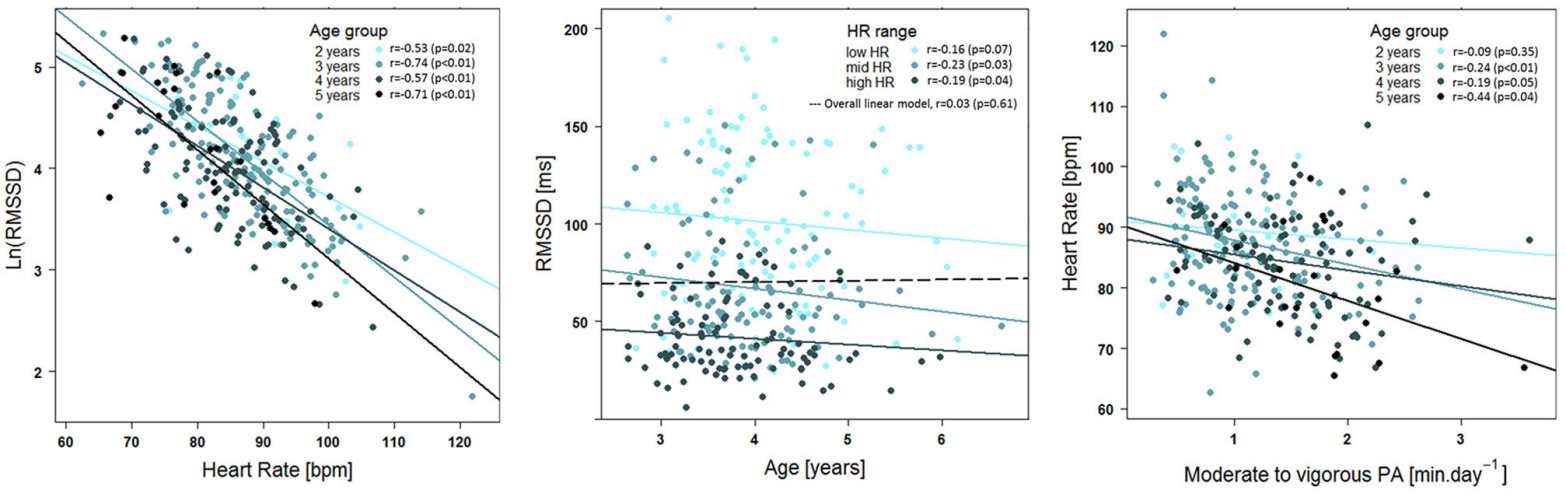
**Raw data scatter plots**. Linear relationship between lnRMSSD and HR for children according to age (marked with different colors) is shown in the left panel. RMSSD [ms^2^] was log transformed to receive a linear relationship with HR. Lines indicate linear regressions for each age group. The weak but significant decline of RMSSD with age for three different HR ranges (low HR: <83 beats.min^−1^ medium HR: 83–91 beats.min^−1^, high HR: >91 beats.min^−1^), reflecting the result of the HR-adjusted mixed linear model for lnRMSSD with age, is illustrated in the middle panel. The overall regression line (dashed line) represents the linear regression of RMSSD vs. age without adjustment for HR. Effect of volume of daily moderate to vigorous physical activity on resting HR for individual age classes is shown in the right panel. Solid lines represent linear regressions for each age group. All plots are based on HRV data from the *high %HF* segments. RMSSD, root mean squared successive differences; ln, natural logarithm; HR, heart rate; PA, physical activity; HF, high frequency.

**Figure 4:**
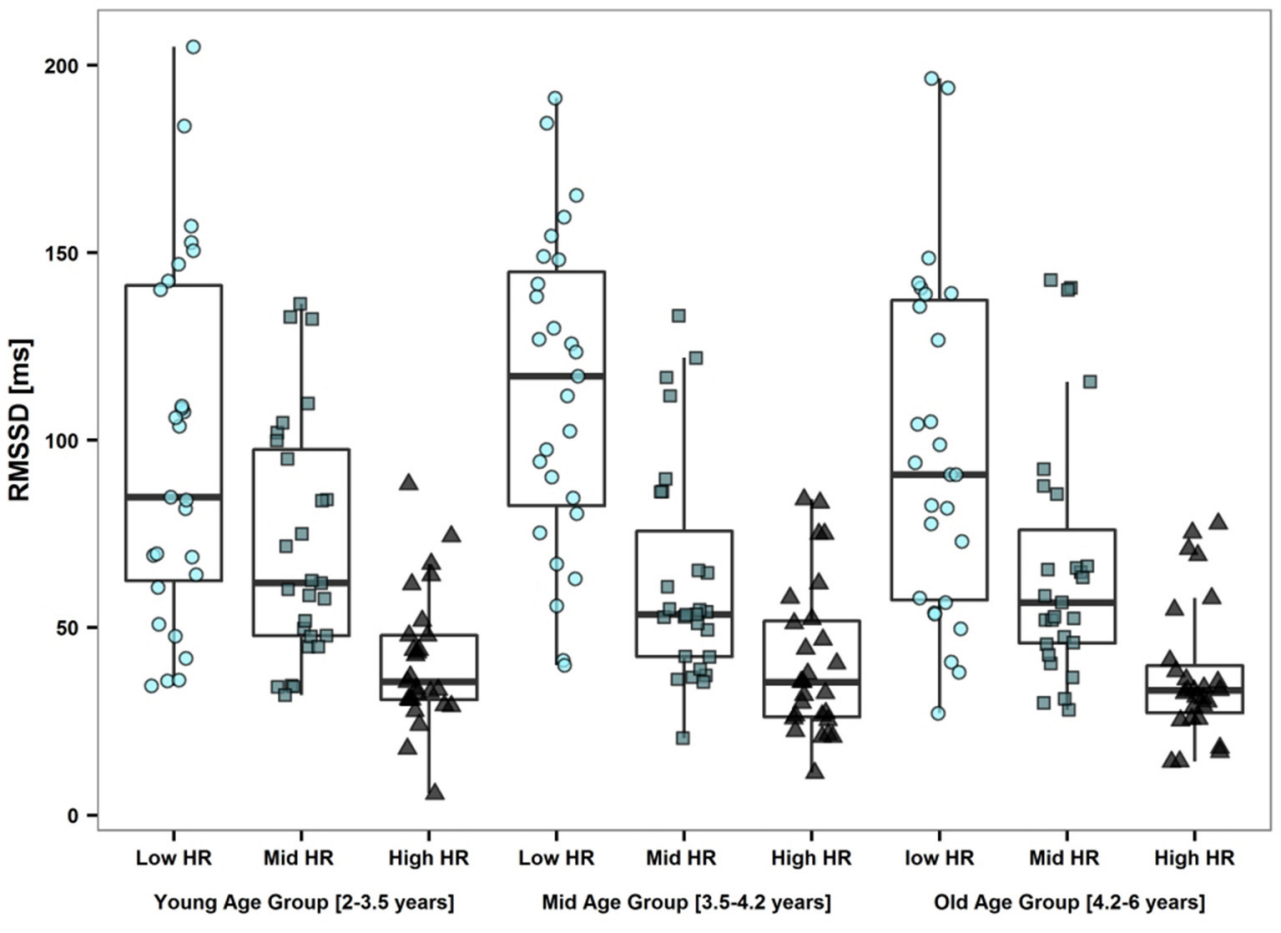
**Subanalysis showing reduced data set**. A total of 243 children were randomly selected, 81 in each age group (young, mid, old) of whom 27 in each HR group (low, mid, high HR group). No effect of age on RMSSD was observed (all *p* > 0.25). Within the three age groups, RMSSD was significantly different between the HR groups (all *p* < 0.01).

Models were conducted for HRV parameters of all three segments. Results of the *15'aSO* segments and *high %HF* segments were comparable, while HRV parameters of the *4-h* segments resulted in non-significant models. Therefore, we only present results from the linear regression models with HRV parameters of the *high %HF* segment (Table 3). RMSSD and SDNN had to be log transformed in order to satisfy the requirement of normal distribution and linearity with HR for the linear models. Inverse effects of age (Figure 2), height and weight were found on HR as well as on RMSSD and SDNN in the models adjusted for HR (all *p* < 0.01). RMSSD and SDNN decreased significantly with increasing age when adjusted for HR, which is illustrated in Figure 3 (middle panel). Pearson correlation coefficients of anthropometric (as well as physical activity) parameters with HR were much greater than with RMSSD or SDNN, except for skinfolds that had the same effect size (Table 4). To avoid redundancy, Spearman correlations were only performed with HRV parameters of the *high %HF* segments. All HRV parameters were strongly correlated with HR. Parameters of PA showed the closest relationship with age (and consequently height and weight), followed by HR and only weak relationships to ln(RMSSD) and ln(SDNN). There was also a weak inverse relationship between skinfolds and parameters of PA. Of note is the correlation coefficient of 0.98 between ln(RMSSD) and ln(SDNN), implying that the selected segments of deep sleep total variability was dominated by variability of successive beats. The dependency of RMSSD on HR is shown in Figure 3 (left panel), with HR explaining 43% of the variance in lnRMSSD (*p* < 0.001). MVPA (Figure 3, right panel) and TPA (and by trend for VPA *p* = 0.053) as well as skinfold thickness were significantly related to HR (all *p* < 0.01) while showing no significant relation with RMSSD and SDNN. HR decreased significantly with increasing TPA and MVPA even after adjusting for the significant effect of age on HR (Table 3, Figure 3, right panel). In the sub-analysis based on the nine groups formed with regard to age and HR, a significant effect of HR (all *p* < 0.01) but no effect of age on RMSSD were observed (all *p* > 0.25, and the same applied to all other HRV parameters; Table 5, Figure 4). Results for frequency domain parameters showed the same effects as for the time domain parameters (HF was consistent with RMSSD and LF with SDNN) and thus, are not reported.

**Table 3:** **Linear regression models of time domain HRV parameters from the High %HF segment**.

	**HR Standardized β**	**ln(RMSSD) Standardized β**	**ln(SDNN) Standardized β**	**DFA alpha 1 Standardized β**
Age	−**0.21^‡^**	−**0.16^‡^**	−**0.15^‡^**	0.09
HR	–	−**0.70^‡^**	−**0.62^‡^**	**0.49^‡^**
Height	−**0.18^‡^**	−**0.13^‡^**	−**0.12^†^**	0.02
HR	–	−**0.68^‡^**	−**0.61^‡^**	**0.48^‡^**
Weight	−**0.13^†^**	−**0.14^‡^**	−**0.14^‡^**	0.02
HR	–	−**0.67^‡^**	−**0.61^‡^**	**0.48^‡^**
BMI	0.06	−0.04	−0.06	0.01
HR	–	−**0.65^‡^**	−**0.59^‡^**	**0.47^‡^**
BMI_Z−score_	0.04	−0.05	−0.06	0.02
HR	–	−**0.65^‡^**	−**0.59^‡^**	**0.47^‡^**
Skinfolds	**0.28^‡^**	−0.01	−0.06	−0.08
HR	–	−**0.63^‡^**	−**0.57^‡^**	**0.49^‡^**
Sex	0.09	0.01	0.02	0.08
HR	–	−**0.65^‡^**	−**0.59^‡^**	**0.48^‡^**
TPA	−**0.17^‡^**	−0.04	0.03	0.01
HR	–	−**0.69^‡^**	−**0.63^‡^**	**0.49^‡^**
Age	−**0.17^‡^**	−**0.15^‡^**	−**0.14^‡^**	0.01
MVPA	−**0.22^‡^**	−0.05	−0.03	0.01
HR	–	−**0.7^‡^**	−**0.63^‡^**	**0.49^‡^**
Age	−**0.14^‡^**	−**0.14^‡^**	−**0.14^‡^**	0.08

*HRV, heart rate variability; HR, heart rate; ln, natural logarithm; RMSSD, square root of the mean squared differences of adjacent RR intervals, SDNN, standard deviation of the RR intervals; DFA, detrended fluctuation analysis; BMI, body mass index; TPA, total physical activity; MVPA, moderate-to-vigorous physical activity. Linear regression models were applied for the dependent variables heart rate (HR), ln(RMSSD) and ln(SDNN) and independent anthropometric parameters entered together with HR, as well as physical activity parameters entered together with HR and age. Bold numbers indicate statistically significant standardized β-coefficients*.

† *p ≤ 0.05*,

‡ *p ≤ 0.01*.

**Table 4:** **Pearson correlation coefficients between anthropometric variables, PA variables and HRV parameters**.

	**HR**	**ln(RMSSD)**	**ln(SDNN)**	**DFA**	**Age**	**Height**	**Weight**	**BMI**	**Sex**	**TPA**	**MVPA**	**Skinfolds**
HR	–											
ln(RMSSD)	−**0.61**^b^	–										
ln(SDNN)	−**0.57**^b^	**0.98**^b^	–									
DFA	**0.47**^b^	−**0.28**^b^	−**0.17**^b^	–								
Age	−**0.27**^b^	0.03	0.03	−0.05	–							
Height	−**0.24**^b^	0.01	−0.01	−0.10	**0.80**^b^	–						
Weight	−**0.17**^b^	−0.03	−0.05	−0.07	**0.60**^b^	0.83	–					
BMI	0.06	−0.05	−0.05	0.03	−**0.17**^b^	−0.07	0.49	–				
Sex	**0.14**^b^	−0.06	−0.06	−0.01	−**0.11**^a^	−**0.21**^b^	−**0.18**^b^	0.01	–			
TPA	−**0.25**^b^	0.10	0.09	−**0.11**^a^	**0.26**^b^	**0.26**^b^	**0.26**^b^	0.06	−**0.14**^a^	–		
MVPA	−**0.30**^b^	**0.11**^a^	0.10	−**0.11**^a^	**0.37**^b^	**0.36**^b^	**0.33**^b^	0.01	−**0.22**^b^	**0.88**^b^	–	
Skinfolds	**0.14**^a^	−**0.14**^a^	−**0.14**^a^	−0.03	−0.09	0.04	**0.31**^b^	**0.52**^b^	**0.26**^b^	−**0.12**^a^	−**0.13**^a^	–

*Abbreviations as in Table 3*.

a *p ≤ 0.05*,

b p ≤ 0.01

**Table 5:** **Sub-analysis: HRV parameters of randomly selected children specifically grouped according to heart rate within age groups**.

	**Young (2–3.5 years)**	**Mid (3.5–4.2 years)**	**Old (4.2–6 years)**	***p*-value**
HR (beats.min^−1^)	85.8 (80.7; 91.6)	86.7 (79.3; 92.0)	85.5 (78.0; 90.9)	0.49^*^
RMSSD (ms)	61.9 (35.8; 95.0)	64.6 (40.4; 97.5)	53.9 (33.3; 82.6)	0.33
SDNN (ms)	46.9 (30.6; 74.3)	53.2. (36.5; 81.2)	44.8 (30.4; 67.7)	0.33
HF power (ms^2^)	1,479 (570; 3,684)	1,679 (818; 3,524)	1,350 (665; 2,775)	0.59
LF power (ms^2^)	236 (106; 557)	296 (121; 751)	221 (100; 574)	0.30
LF/HF	0.19 (0.11; 0.26)	0.17 (0.11; 0.30)	0.15 (0.10; 0.27)	0.48
Total Power (ms^2^)	1,947 (711; 4,668)	2,225 (974; 5,100)	1,711 (766; 3,781)	0.71
DFA alpha 1	0.55 (0.45; 0.63)	0.54 (0.46; 0.63)	0.55 (0.45; 0.64)	0.81

*Abbreviations as in Table 3*.

*Medians (interquartile ranges) are shown. p-values are from Kruskal–Wallis testing. A total of 243 children were randomly selected, 81 in each age group (young, mid, old) of whom 27 in each HR group (low, mid, high HR group)*.

* *Please note there is no difference in HR between the three age groups because groups were specifically formed with regard to HR*.

## Discussion

The results of the present study show a decrease in HR with increasing age and PA, an increase with skinfold thickness, and no increase in HRV parameters including the commonly used markers of vagal tone. When HRV parameters were adjusted for HR there was a decrease in RMSSD and SDNN with age. This is in accordance with one recent study (Gasior et al., 2015) but in contrast to many previous studies (Finley and Nugent, 1995; Goto et al., 1997; Villa et al., 2000; Silvetti et al., 2001; Michels et al., 2013). The adjustment with HR is based on the assumption that intrinsic heart rate declines with age due to the growth of the heart (Cumming and Mir, 1970). Adjusting HRV parameters for HR in children of an age range where large declines in HR occur has a major impact on HRV results and their interpretation (Monfredi et al., 2014; Gasior et al., 2015). Further, there was no association between HR-adjusted HRV parameters reflecting vagal tone with sex, BMI, skinfold thickness, and PA. However, HR itself was related to skinfold thickness and inversely related to PA. Further, by performing a sub-analysis based on groups comparable either with regard to age or HR, we attempted to overcome the problem of HRV dependency on HR. No differences in HRV parameters were observed in the age groups with similar HR (Table 5), despite a higher amount of PA in the older age groups (Table 4). These results do not support an age-related increase of vagal tone in young children, contrary to what has been suggested previously. Based on the methods used in our study, we cannot identify the exact reasons of the age-related decrease in heart rate, however we propose that structural and morphological changes of the heart during growth may be responsible.

There are a number of previous studies that have assessed HRV in young children. However, only some of them have reported nocturnal HRV measurements (Finley and Nugent, 1995; Goto et al., 1997; Massin and von Bernuth, 1997). Two studies have described an increase in HRV parameters reflecting vagal tone (RMSSD, HF, and to a lesser extent SDNN and TP) from infancy to age 6 years and a slow decrease of these parameters thereafter (Finley and Nugent, 1995; Goto et al., 1997). Several more studies have found an increase in HRV markers of vagal tone in children older than 6 years (for a review see Eyre et al., 2014). Most of these studies (Finley and Nugent, 1995; Goto et al., 1997; Michels et al., 2013) did not control for HR despite the recognized importance of correcting HRV parameters with HR (Sacha and Pluta, 2008; Billman, 2013). The only study that has adjusted HRV parameters for prevailing HR has found no change in HRV parameters with age in children aged 6–13 years (Gasior et al., 2015). In accordance with our results, they found a strong decrease of HR with age and also an age-related decrease in HR-adjusted HRV parameters. In another cohort of 460 boys and girls aged 5–12 years a significant increase in RMSSD and HF was found with age without adjusting models for HR (Michels et al., 2013). After adjustment with HR, the authors also found RMSSD and HF power to be inversely related to age (pers. Comm.) which is in accordance with our and Gasior and colleagues' results (Gasior et al., 2015). A further study found both age and HR to be independently related to HRV parameters as well as to each other (Massin and von Bernuth, 1997). They pointed out the difficulty of a correct interpretation of autonomic nervous activity because of an interdependence between age, HR, respiration frequency, blood pressure, and HRV.

It is likely that in growing children, HR decreases as a consequence of different factors such as the growing heart which grows proportionally to height; (St John Sutton et al., 1982; O'Leary et al., 2015), larger total blood volume and increasing blood circulation time (Morse et al., 1947). An inverse relationship between body mass and HR has been found across different mammal species [for summary see Dobson et al. (Dobson, 2003)]. HRV has been found to be inversely related to HR, with larger R-R intervals allowing for larger variation (Billman, 2013; Dvir et al., 2013; Monfredi et al., 2014). The dependency of HRV on HR has recently been described showing that HRV is inextricably linked to HR in an exponential manner (Zaza and Lombardi, 2001), for which biophysical properties have been suggested to be responsible (Monfredi et al., 2014). The importance of correcting for prevailing HR to reflect cardiac autonomic activity more closely has been pointed out by a study on different autonomic interventions (Billman, 2013). Taking this into account, values of HRV parameters may not reflect activity of the cardiac autonomic nervous system across different age ranges in growing children. Rather, the often documented increase in HRV parameters with age mainly reflects the age-related decrease in HR, which, between age 2 and 6 years, has been found to be ~20 beats.min^−1^ (Goto et al., 1997; Fleming et al., 2011; O'Leary et al., 2015).

When HRV is used as a marker of the autonomic nervous activity, correction for HR has to be considered. HRV parameters have been shown to reflect autonomic nervous activity in many previous studies (Pomeranz et al., 1985; Pagani et al., 1986; Malik and Camm, 1993; Goldberger et al., 2001). More recently, studies have suggested the necessity of correcting HRV for HR (Billman, 2013; Sacha, 2013; Monfredi et al., 2014; Gasior et al., 2015). There is no consensus as yet on whether HR correction should be applied or not, probably because the adequate answer depends on the study design, the study population and the research question. While the strong relation between HRV and HR has been shown to be partly due to mathematical (Sacha and Pluta, 2008; Sacha, 2014) and biophysical properties (Zaza and Lombardi, 2001; Monfredi et al., 2014), part of this association is due to the concurrent influence of the autonomic nervous system on both parameters (i.e., increase in vagal activity both increases RMSSD and decreases HR) and correcting with HR could therefore remove observed differences in RMSSD. In our study population of young children between 2 and 6 years, in whom growth is likely to have a substantial effect on structural and morphologic changes of the heart, failing to correct HRV for heart rate may lead to erroneous interpretation of vagal activity with regard to age. Therefore, we suggest that there is no general rule with regard to heart rate adjustment of HRV parameters. We propose that in the absence of knowledge on intrinsic heart rate the interpretation of vagal activity based on “established markers of vagal tone” is risky, to say the least, particularly in populations with large variances with regard to heart structure and/or morphology. The only study that determined intrinsic HR under double blockade of the cardiac autonomic nervous system with propranolol and atropine in 103 infants and children aged 0–16 years with mild to moderate heart defects has found an age related decline in intrinsic HR (Cumming and Mir, 1970). Intrinsic HR was ~20 beats.min^−1^ higher than control resting heart rate without any blockade at all ages. The authors concluded that the decline in HR was not due to changes in autonomic function. It was suggested that age related changes in the frequency of depolarization in pacemaker tissue were responsible, such as changes in sino-atrial node membrane ion flux or permeability, or alterations in location of the predominant pacemaker cells within the node (Rowland, 2004). Unfortunately, there are no studies on age-related changes in intrinsic HR in healthy children.

We found physical activity to be inversely related to HR but not to HRV parameters in our models adjusted for HR. This is in accordance with what was found in a comparable study in boys, but not in girls (Michels et al., 2013). Numerous studies have assessed the influence of obesity or body composition on HRV in children and adolescents (for a review see Eyre et al., 2014). However, the only study who investigated younger children comparable to our cohort found no association between BMI or percent body fat with HRV parameters (Michels et al., 2013). We observed that increasing skinfold thickness was related to HR, but not to HRV parameters in models adjusted for HR. In older children, several studies have documented reduced HRV markers of vagal tone and increased HR in overweight and obese children compared to normal children (Rabbia et al., 2003; Vanderlei et al., 2010; Altuncu et al., 2012), and more so in obese children with larger amount of central fat (Soares-Miranda et al., 2011).

The close correspondence of deep sleep segments identified by two different methods, one semi-automatically (by decrease in HR und cessation of activity) and one by an algorithm based on high percentage of HF power indicates a reliable identification of deep sleep. The systematic difference between the two segments was most likely due to the later occurrence of the automatically detected segment with an already lower HR. In contrast, HRV during the *4-h* segment was more weakly related to those of deep sleep. The reason for this may be the greater proportion of REM-sleep in the second half of the night (Montgomery-Downs et al., 2006), which is supported by the higher values of SDNN, LF power, and total power. A 4-h segment of the second half of the night is therefore not suitable as it is very heterogeneous and greatly confounded by individual sleep architecture. On the other hand, our method to identify deep sleep phases by high percentage of HF power (corresponding with a low LF/HF ratio) has already been suggested for adults (Camm, 1996). It does not require additional equipment on top of an ECG monitor and does not depend on transition time from sleep onset to deep sleep.

A limitation of the present study is the missing information on true intrinsic HR of our subjects. Our hypothesis that there is a growth-related decrease in HR is based on the only existing study that has determined intrinsic HR under vagal and sympathetic blockade in children, and more general literature that has related HR to heart size. Therefore, we cannot define the development of vagal activity with age but we stress that interpretation of cardiac autonomic nervous system activity greatly depends on the assumption of the origin of HR decline with age (i.e., that either there is a growth-related decrease of intrinsic HR or that HR declines as a consequence of increased vagal activity). Further, the present results are based on cross-sectional data. Longitudinal data over this age range would have reduced the data variance, however, longitudinal data would most likely also show a reduction in HR and the directly linked increase in vagal markers of HRV, without giving any further explanation as to what the origin of HR decline is. Measurements of skinfold thickness only provide an approximation of body composition. However, they were chosen because of non-invasiveness and higher precision than BMI.

Strengths of the present study are the automatic identification of a deep sleep segment providing stationary HRV data undisturbed by environmental stimulants and with regular respiration frequency which could be an optimal method to assess HRV in young children, and secondly, the adjustment for HR in models relating anthropometric and physical activity parameters to HRV markers of vagal tone.

In conclusion, we found no increase of standard HRV parameters with age, however, when adjusted for HR, there was a significant decrease of HRV parameters with increasing age, in accordance with one previous study (Gasior et al., 2015). The age-related decrease of HR, observed across subjects in our cross-sectional data sample, cannot simply be interpreted as an increase in vagal activity. Instead, at least some of the decrease in HR may be related to growth with an increase in heart, vessel size, and blood volume leading to a decrease in intrinsic heart rate. Whether the observed decrease in HR-adjusted HRV parameters reflects a true decrease in vagal activity between age 2 and 6 years remains to be elucidated in longitudinal studies using direct measurement of vagal activity. Thus, in the absence of available measurements of intrinsic heart rate, HRV changes during growth have to be interpreted with caution.

## Author contributions

DH, PE, SK, TRa, PA, MW, JP, OJ, SM: designed research of the substudy; DH, PE, TRa, TRu, AW, PA, SK performed data analyses; DH, PE: performed statistical analyses; DH, PE, TRa, SK, PA: wrote and commented the manuscript; NM, TK, KS, CL, ES, AZ, AA, AW, TRa: contributed to data collection. All authors approved the final version of the manuscript.

### Conflict of interest statement

The authors declare that the research was conducted in the absence of any commercial or financial relationships that could be construed as a potential conflict of interest.
